# Concomitant autoimmunity and late cancers in adult-onset immunodeficiency due to neutralizing anti-IFN-γ autoantibodies

**DOI:** 10.3389/fimmu.2025.1526439

**Published:** 2025-04-17

**Authors:** Wan-Ting Tsai, Chih-Yun Cheng, Hsin-Yun Sun, Bei-Chia Guo, Ying-Chieh Chiang, Chiao-Feng Cheng, Yi-Hua Pan, Un-In Wu, Jann-Tay Wang, Wang-Huei Sheng, Aristine Cheng, Yee-Chun Chen, Shan-Chwen Chang

**Affiliations:** ^1^ Department of Internal Medicine, National Taiwan University Hospital and National Taiwan University College of Medicine, Taipei, Taiwan; ^2^ Department of Internal Medicine, Taipei City Hospital, Zhongxiao Branch, Taipei, Taiwan; ^3^ Department of Internal Medicine, National Taiwan University Hospital, Hsinchu Branch, Hsinchu, Taiwan

**Keywords:** anti-interferon-γ autoantibody, autoimmunity, secondary immunodeficiency, urothelial carcinoma, papillary thyroid carcinoma, autoimmune thyroiditis

## Abstract

**Background:**

Opportunistic intramacrophagic infections are well-characterized in adult-onset immunodeficiency associated with neutralizing anti-IFN-γ autoantibodies (nAIGA).

**Objective:**

Concomitant autoimmune and neoplastic diseases are rarely described.

**Methods:**

This study included 50 patients diagnosed with adult-onset immunodeficiency due to nAIGA between 2014-2024. Thirty-three were retrospectively included before January 2022, and 17 out of 295 screened patients were enrolled prospectively since January 2022. Ten patients were excluded due to missing records. All patients had regular follow-ups; anti-IFN-γ titers, autoimmune markers and cancer survey were conducted according to the primary physician’s evaluation.

**Results:**

The median age at diagnosis of adult-onset immunodeficiency was 57 years, and 53% were men. Malignancy occurred in 25%; genitourinary cancer predominated (n=4). Most (93%) patients had at least one positive autoimmune marker. Fifty-eight percent of patients were diagnosed with concomitant autoimmune diseases, and women (65%) predominated. Anti-nuclear antibody was positive in 61%, lupus anticoagulant in 50%, whilst autoimmune thyroiditis markers in 43%. Twenty-two percent of patients required long-term immunomodulation including biologic agents such as rituximab and daratumumab. Three patients (8%) died after a median interval of 9.4 years due to sepsis (n=2) and aggressive urothelial cancer (n=1). Most patients had decreasing nAIGA titers over time; two outliers with persistently high neutralizing antibodies developed late-onset malignancies.

**Conclusion:**

Adult-onset immunodeficiency due to nAIGA is a syndrome associated with concomitant autoimmunity. Chronic infection and autoimmune-mediated inflammation may foster neoplastic changes, but the underlying mechanism is still undetermined. Autoimmune disease and cancer surveillance for patients with nAIGA is advised.

## 1 Introduction

Neutralizing anti-IFN-γ autoantibodies (nAIGA) are recognized as an uncommon etiology of adult-onset immunodeficiency. These autoantibodies are typically described as standalone autoantibodies that neutralize IFN-γ, inhibit the STAT1 pathway, and result in defective macrophage function (1, 2). Due to defective killing of phagocytosed microbes, granuloma formation, and T-helper-1 (Th1) activation, patients with nAIGA are prone to disseminated intracellular infections due to mycobacteria, *Salmonella, Burkholderia*, *Talaromyces marneffei*, and *Cryptococcus* spp (3). However, like other inborn errors of immunity, defective IFN-γ immunity may not only result in defective immunity against certain infections, but also dysregulated immunity against self-antigens and cancerous cells (4).

Demographically, patients with nAIGA are also similar to patients with other autoimmune conditions; they are more often female (60-64% in Thailand and Taiwan, 91% in the USA) and middle-aged (median 45 to 50 years old) at disease onset (3, 5). However, autoimmune diseases and malignancies have rarely been described in detail in case reports or case series of patients with nAIGA (**Supplementary Table S1**). The manifestations of nAIGA, such as reactive lymphadenopathies, have been compared and contrasted with hematological diseases (6, 7) but lymphadenopathies in this condition can also mimic autoimmune or non-hematological neoplastic changes (8, 9).

Due to the initial severity and multiorgan involvement of infections, symptoms of concomitant autoinflammatory or autoimmune disease, with the exception of skin manifestations such as Sweet’s syndrome or generalized pustular psoriasis, are underappreciated (10). In addition, since patients with nAIGA are typically considered immunocompromised and managed by infectious disease physicians rather than rheumatologists, serological markers and treatment for other autoimmune conditions are infrequently sought for. Late recurrence of lymphadenopathies or rebound nAIGA titers may be treated presumptively as relapsed infectious disease without prompt survey for incidental cancers.

The aims of the present study were to describe the frequency of concomitant autoimmune and neoplastic disease, and to describe the challenging management of concomitant immunomodulation and chemotherapy in the setting of disseminated infections.

## 2 Materials and methods

### 2.1 Study setting and population

This study was conducted between 2014-2024 at the National Taiwan University Hospital (NTUH), a 2500-bed tertiary university hospital in Taipei. Patients visiting NTUH and its branches received nAIGA testing if they had unexplained or recurrent opportunistic infections suggestive of adult-onset immunodeficiency and were seronegative for human immunodeficiency virus. The patients would receive assessment for concomitant autoimmune, neoplastic diseases, and opportunistic infections during admissions typically for fever, lymphadenopathy or weight loss. They were all under the long-term care of physicians of the infectious diseases department. The patients were followed on a monthly basis whilst on antimicrobials or every 3 months during periods of disease quiescence until loss to follow-up, death, or August 31^st^ 2024, whichever came first.

Since January 2022, patients were enrolled in a prospective study and those who gave informed consent had blood routinely sampled for anti-IFN-γ titers at 3-6 monthly intervals. Plasmas or sera were stored in cryovials at -80 deg C until use. The results of longitudinal anti-IFN-γ titer analyses were conducted retrospectively using banked plasmas where available. The study was approved by the Institutional Review Board committee of the National Taiwan University Hospital (201412163RIND, 202112132RIND, 202201034RINA).

### 2.2 Outcome measures

Clinical data were retrospectively and manually collected from medical records. Clinical laboratory investigations included lymphocyte surface markers and immunoglobulin titers at the time of diagnosis of adult-onset immunodeficiency due to nAIGA, and inflammatory biomarkers and rheumatological autoantibody analyses at the time of diagnosis of concomitant autoimmune disease. All the patients received screening for common rheumatological autoantibodies, but the extent and frequency of testing were decided by the primary physician. Patients also received a fludeoxyglucose-18-positron emission tomography (FDG-PET) scan for monitoring residual disease or inflammatory hot-spots when indicated, typically on an annual basis when maintained on oral antimicrobial agents.

Opportunistic infections such as nontuberculous mycobacterial (NTM) or mold infection were defined based on IDSA guidelines (11, 12). Autoimmune diseases such as autoimmune thyroid disease, mixed connective tissue disease, and undifferentiated connective tissue disease (UCTD) were defined according to established criteria and by an attending rheumatologist (13–16).

### 2.3 Detection of anti-cytokine autoantibodies

Plasma or serum samples were screened for binding autoantibodies against routine targets (e.g. IFN-γ, GM-CSF, IL-23, IFN-α) in a multiplex particle-based assay, in which magnetic beads with differential fluorescence were covalently coupled to recombinant human proteins (2.5 μg/reaction). The beads were combined and incubated with 1:100 diluted serum/plasma samples for 30 minutes. Each sample was tested at least once in duplicate. The beads were then washed and incubated with PE-labelled goat anti-human IgG (1 μg/mL, eBioscience Catalog # 12-4998-82) for an additional 30 minutes. The beads were then washed again and run on a Luminex instrument in a multiplex assay (17).

### 2.4 Bioactivity of anti-cytokine-binding autoantibodies by inhibition of STAT phosphorylation

The neutralizing activities of the anti-IFN-γ autoantibodies were determined by assessing STAT1 phosphorylation, after stimulation with the IFN-γ in the presence of 10% healthy control or patient plasma using human HEK-Blue™ reporter cell lines (Invivogen, Catalog #hkb-ifng) according to the manufacturer’s instructions (17). Plasmas or sera were serially diluted in the presence of 1ng/mL of recombinant human IFN-γ (R&D Systems Inc., Minneapolis, Catalog # 285-IF) and the neutralizing titer was defined as the first dilution of plasma that no longer inhibited STAT1 signaling.

### 2.5 Statistical analyses

All the statistical analyses were conducted using the Stata software package, version 17.0 (StataCorp). Categorical variables were assessed using the chi-square test or Fisher’s exact test, while continuous variables were compared using the Student’s t-test or the Wilcoxon-Mann–Whitney test. Graphs to examine the relationship between binding or neutralizing autoantibody titers and the follow-up time were drawn using Graphpad (Prism 10.0). All the statistical tests were two-sided, and variables with P-value < 0.05 were considered to be statistically significant.

## 3 Results

**Figure 1:**
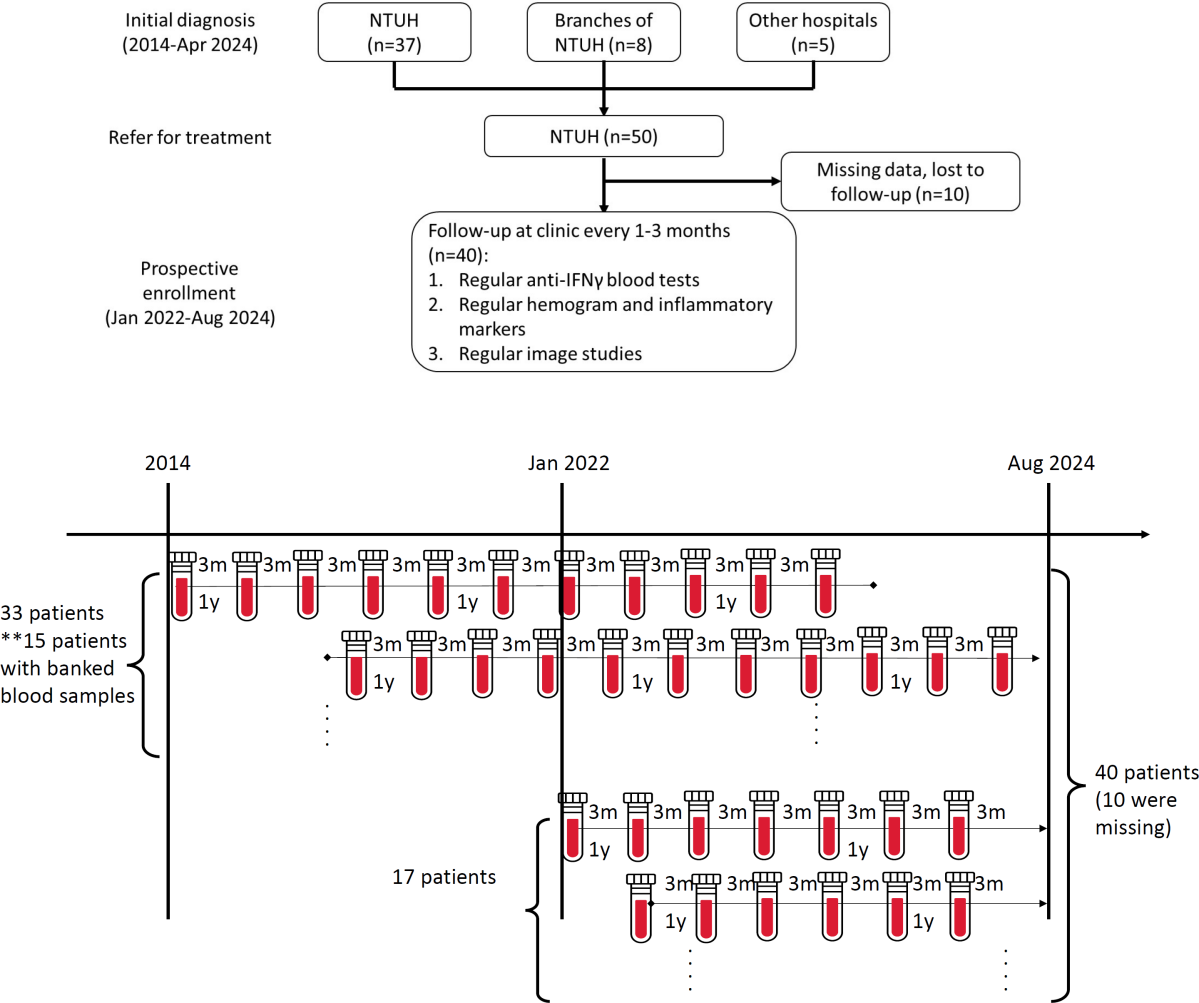
(Upper panel) Study flow; (Lower panel) The timeline and blood sampling period.

During the study period (2014–2024), 50 patients with adult-onset immunodeficiency secondary to nAIGA at NTUH were identified by retrospective chart review, 33 of whom were diagnosed before January 2022. Between Jan 2022- Aug 2024, 295 patients were screened for anti-cytokine autoantibodies, and 17 were newly-diagnosed with nAIGA. After excluding ten patients due to missing records or follow-up at other hospitals, a total of 40 patients were analyzed, fifteen of whom had banked plasmas prior to 2022 for longitudinal follow-up (**Figure 1A**). Of 27 HIV-negative patients with disseminated NTM bacteremia diagnosed during 2022-2024, the prevalence of nAIGA was 63% (17/27).

Patient demographics are presented in **Table 1**. The patients were on average 57 years old and 53% were men, and had been followed for a median of 4.4 years. Patients had mildly elevated serum IgG and IgG-1 levels at the time of nAIGA diagnosis (**Supplementary Table S2**). The most common comorbidity was gastroesophageal reflux disease, gastritis or gastric ulcers (25, 63%), followed by malignancies (10, 25%). During follow-up, four (10%) out of the 10 patients developed new gynecologic or genitourinary malignancies, making them the most common type in this study. All the patients with positive nAIGA had disseminated mycobacterial infections, mostly with lymph nodes (83%), lung (43%), bone (40%), and blood (30%) involvement. Only three (8%) patients died during follow-up. The causes of death were aspiration pneumonia in an 80-year-old man, severe influenza complicated with invasive pulmonary aspergillosis in a 45-year-old woman, and stage IV urothelial cancer in a 67-year-old woman. Most (93%) of the patients tested positive for common autoantibodies (**Table 2**), and 58% (23/40) of the patients were diagnosed with concomitant autoimmune diseases after the diagnosis of adult-onset immunodeficiency. Patients with concomitant autoimmune diseases were younger (median age 54 vs. 59) and predominantly female (65% vs. 24%) (both p<0.05) (**Table 1**). The mortality rate was not different among patients with or without concomitant autoimmune diseases (p=0.25).

**Table 1:** Characteristics of 40 patients with neutralizing anti-IFN-γ autoantibodies in relation to concomitant autoimmune disease (AiD).

Variables	Overall	With AiD	Without AiD	p value
Basic profile				
Patient number, n	40	23	17	NA
Age, year (median [IQR])	56.5 (52.5-63.5)	54 (42-58)	59 (56-65)	0.019
Men, n (%)	21 (52.5)	8 (34.8)	13 (76.5)	0.012
Positive autoimmune markers, n (%)	37 (92.5)	23 (100.0)	14 (82.4)	0.069
Titer of binding anti-IFN**-**γ autoantibody, median (IQR)	9775 (8964-11862)	9857 (8452-11999)	9693 (9308-10310)	0.775
Titer of neutralizing anti-IFN-γ autoantibody^a^, median (IQR)	1:64 (1:16 - 1:256)	1:64 (1:16 – 1:256)	1: 64 (1:64- 1:128)	0.278
Any comorbidities, n (%)				
GERD or gastric ulcer	25 (62.5)	15 (65.2)	10 (58.8)	0.749
Chronic hepatitis B infection	5 (12.5)	2 (8.7)	3 (17.6)	0.634
Malignancy	10 (25.0)	8 (34.8)	2 (11.8)	0.145
Hodgkin’s lymphoma	1 (2.5)	1 (4.3)	0 (0.0)	1.000
Papillary thyroid cancer	2 (5.0)	2 (8.7)	0 (0.0)	0.499
Genitourinary/gynecologic	4 (10.0)	3 (13.0)	1 (5.9)	0.624
Other malignancies^b^	4 (10.0)	2 (8.7)	2 (11.8)	1.000
Hypertension	9 (22.5)	4 (17.4)	5 (29.4)	0.456
Diabetes	9 (22.5)	4 (17.4)	5 (29.4)	0.456
COPD	1 (2.5)	0 (0.0)	1 (5.9)	0.425
Congestive heart failure	0 (0.0)	0 (0.0)	0 (0.0)	NA
Chronic hepatitis C	2 (5.0)	1 (4.3)	1 (5.9)	1.000
Chronic kidney disease	3 (7.5%)	2 (8.7%)	1 (5.9%)	1.000
History of opportunistic infections, n (%)				
Culture-proven mycobacterial infection	37 (92.5)	22 (95.7)	15 (88.2)	0.565
Nontuberculous mycobacteria (NTM)	35 (87.5)	22 (95.7)	13 (76.5)	0.144
Both tuberculosis and NTM	2 (5.0)	0 (0.0)	2 (11.8)	0.174
*Organs involved by mycobacteria*				
Lymph nodes	33 (82.5)	19 (82.6)	14 (82.4)	1.000
Lung	17 (42.5)	12 (52.2)	5 (29.4)	0.202
Bone	16 (40.0)	8 (34.8)	8 (47.1)	0.522
Blood	12 (30.0)	9 (39.1)	3 (17.6)	0.179
Skin and soft tissue	15 (37.5)	8 (34.8)	7 (41.2)	0.749
Liver, spleen, intestines	7 (17.5)	4 (17.4)	3 (17.6)	1.000
Meninges	3 (7.5)	2 (8.7)	1 (5.9)	1.000
Non-mycobacterial pathogens				
*Herpesviridae* HSV/VZV	14 (35.0)	11 (47.8)	3 (17.6)	0.092
Salmonellosis	13 (32.5)	8 (34.8)	5 (29.4)	1.000
Fungal infection	9 (22.5)	7 (30.4)	2 (11.8)	0.256
*Candida* species	4 (10.0)	3 (13.0)	1 (5.9)	0.624
Mold species	3 (7.5)	3 (13.0)	0 (0.0)	0.248
*Cryptococcus*	1 (2.5%)	1 (4.3%)	0 (0.0%)	1.000
Other fungi^c^	4 (10.0%)	2 (8.7%)	2 (11.8%)	1.000
Outcomes, median (IQR)				
Follow-up duration, year	4.36 (2.47-7.00)	5.90 (3.03-7.13)	3.18 (1.49-5.94)	0.094
Neutralizing AIGA-associated events, n	4 (2-8.5)	6 (2-13)	3 (2-6)	0.056
Operations, n	1 (0-2)	1 (0-2)	1 (0-1)	0.793
Admissions, n	3 (1-7)	6 (1-11)	3 (1-4)	0.035
Death, n (%)	3 (7.5)	3 (13.0)	0 (0.0)	0.248

a The binding titers were expressed as raw fluorescence intensities on the first plasma or serum sample tested.

b Other malignancies: hepatocellular carcinoma (1), hepatocellular carcinoma and prostate cancer (1), colon cancer (1), lung cancer (1), breast cancer and endometrial cancer (1, overlapped with the column “Genitourinary and gynecologic malignancies”).

c Other fungi: *Talaromyces* spp. (2), *Pneumocystis* jirovecii (1), *Trichosporon* spp. (1).

AIGA, anti-interferon-γ antibody; COPD, chronic obstructive pulmonary disease; ED, emergency department; GERD, gastroesophageal reflux disease; HSV, herpes simplex virus; IQR, interquartile range; NTM, nontuberculous mycobacteria; VZV, varicella-zoster virus.

**Table 2:** Characteristics of 37 patients with positive autoimmune markers.

Variables	Total
Basic profile, n (%)	
Women	19 (51.3)
Concomitant AiD	23 (62.2)
The development of positive autoimmune markers	
Before diagnosis of nAIGA Interval, days (median [IQR])	11 (29.7) 228 (60-3735)
At diagnosis of nAIGA	17 (46.0)
After diagnosis of nAIGA Interval, days (median [IQR])	9 (24.3) 696 (137-1686)
Neutralizing AIGA-associated events, median (IQR)	4 (2-8)
Operations	1 (0-2)
Admissions	3 (1-7)
Autoimmune markers positivity, positive/tested patients (%)	
Number of autoimmune markers, median (IQR)	2 (1-4)
Patients with >2 autoimmune markers	18/37 (48.7)
Anti-nuclear antibody	22/36 (61.1)
Lupus anticoagulant (dRVVT)	15/30 (50.0)
Any markers of autoimmune thyroiditis	16/37 (43.2)
Anti-thyroid peroxidase (Anti-TPO)	10/31 (32.3)
Anti-TSH receptor antibodies (TBII)	5/22 (22.7)
Anti-thyroglobulin (Anti-TG)	8/31 (25.8)
Anti-cardiolipin IgM	7/27 (25.9)
Anti-phospholipid IgM	6/25 (24.0)
Anti-phospholipid IgG	5/24 (20.8)
Anti-dsDNA	5/30 (16.7)
Anti-cardiolipin IgG	2/28 (7.1)
Anti-SSa	3/22 (13.6)
Anti-SSb	1/22 (4.6)
Rheumatoid factor	4/32 (12.5)
Anti-β2-Glycoprotein IgG (B2GP1)	0/18 (0.0)
Autoimmune diseases, n (%)	
Undifferentiated connective tissue disease	11 (29.7)
Autoimmune thyroid disease	9 (24.3)
Hashimoto’s disease	4/34 (11.8)
Graves' disease	1/34 (2.9)
Antiphospholipid syndrome	2 (5.4)
Systemic lupus erythematosus	1 (2.7)
Rheumatoid arthritis	1 (2.7)
Autoimmune diseases, n (%)	
Sjogren’s syndrome	3 (8.1)
Mixed connective tissue disease	1 (2.7)
IgG4-related disease	1 (2.7)
Immunosuppressants, n (%)	
Initial corticosteroids use	24 (64.9)
Maintenance corticosteroids use	5/24 (20.8)
Dose of maintenance corticosteroids, mg (median [IQR])	10 (7.5-12.5)
Pulse cyclophosphamide therapy	3 (8.1)
Pulse corticosteroid therapy	1 (2.7)
Hydroxychloroquine	22 (59.5)
DMARDs (≥2)	8 (21.6)
Biologic agents	12 (32.4)
Multiple biologic agents (≧2)	5/12
Daratumumab	6 (16.2)
Rituximab	4 (10.8)
Others^a^	8 (21.6)
Outcomes, n (%)	
Seroreversion of autoantibodies	7 (18.9)
Without additional follow-up testing	15 (40.5)
Death	3 (8.1)

a Belimumab (n=3), baricitinib (n=3), upadacitinib (n=2), abatacept (n=1).

AiD, autoimmune disease; AIGA, anti-interferon-γ antibody; anti-β2GP1, anti-beta2-glycoprotein 1; anti-SSa, anti-Sjogren’s Syndrome A; anti-SSb, anti-Sjogren’s Syndrome B; anti-TG, anti-thyroglobulin; anti-TPO, anti-thyroid peroxidase; DMARD, disease-modifying antirheumatic drugs; dRVVT, diluted Russell Viper venom time; IQR, interquartile range; TBII, thyroid stimulating hormone-binding inhibitor immunoglobulin; TSH, thyroid stimulating hormone.

Among the 37 (93%) patients with positive autoimmune markers, 18 (49%) had multiple autoantibodies (**Table 2**). The most common autoimmune marker was anti-nuclear antibody (ANA) (22/36, 61%), followed by lupus anticoagulant (15/30, 50%) and any one marker of autoimmune thyroiditis, namely anti-thyroid peroxidase, anti-thyroglobulin, or anti-TSH-receptor antibody (16/37, 43%). The most common rheumatological diagnosis was autoimmune thyroiditis (24%). The prevalence of autoimmune thyroiditis might be underestimated, since only 43% (16/37) of patients were tested for seromarkers of autoimmune thyroiditis. Of those 9 patients diagnosed with autoimmune thyroiditis, the majority were clinically euthyroid, only 3 required levothyroxine, and none required radioactive iodine or anti-thyroid medications. Unclassified connective tissue disease (UCTD) was the most common ICD-10 diagnosis given to 30% of patients with positive serological markers and symptoms not meeting specific criteria for other rheumatological conditions.

To manage background autoimmunity, corticosteroids were initiated in 65% (24/37) of the patients, but only 21% (5/24) of these patients were maintained on corticosteroids. Hydroxychloroquine was prescribed for 59% of the patients, and 22% received two or more disease-modifying antirheumatic drugs (DMARDs). Biological agents, including rituximab and daratumumab, were prescribed to 32% of patients for the control of nAIGA or concomitant autoimmune diseases, and 5 out of 12 required multiple biologic agents. Only 19% (7/37) of patients achieved seroreversion of autoantibodies. One patient with concomitant rheumatoid arthritis (RA) who received abatacept, leflunomide and methotrexate, developed acute exacerbation of chronic hepatitis B virus infection 3 years after the diagnosis of RA. Another patient with autoimmune thyroiditis and UCTD received hydroxychloroquine, leflunomide, belimumab, and daratumumab; she developed pulmonary aspergillosis 2.5 years after control of disseminated nontuberculous mycobacterial disease. Both patients were managed at the clinic with oral agents. In contrast, a patient without immunomodulation, died of invasive pulmonary aspergillosis after critical influenza. The prescription of immunosuppressant did not significantly increase the already high rate of infectious complications. Among the patients with or without maintenance immunosuppressant, the overall recurrent infection rates were 72% (18/25) and 67% (8/12), respectively, after initial diagnosis of nAIGA (p=1.00). Nevertheless, both patients with non-tuberculous mycobacteremia and infection by environmental molds had higher numbers of positive autoimmune markers than those without (p=0.006 and p= 0.007 respectively) and mortality also trended with a higher burden of concomitant autoimmunity (p=0.051) (**Table 3**).

**Table 3:** Predictive contributions of the number of positive autoantibodies and autoimmune disease in patients with neutralizing anti-IFNγ- autoantibodies.

	Mycobacteremia		*P*	Mold infection		*P*	Death		*P*
	With	Without		With	Without		With	Without	
Number of positive autoantibodies	3.58 ± 1.88	2.00 ± 1.44	0.006	5.00 ± 1.73	2.27 ± 1.57	0.007	4.33 ± 0.58	2.32 ± 1.70	0.051
Concomitant autoimmune disease	75.0% (9/12)	50.0% (14/28)	0.179	100.0% (3/3)	54.1% (20/37)	0.248	100.0% (3/3)	54.1% (20/37)	0.248

**Figure 2:**
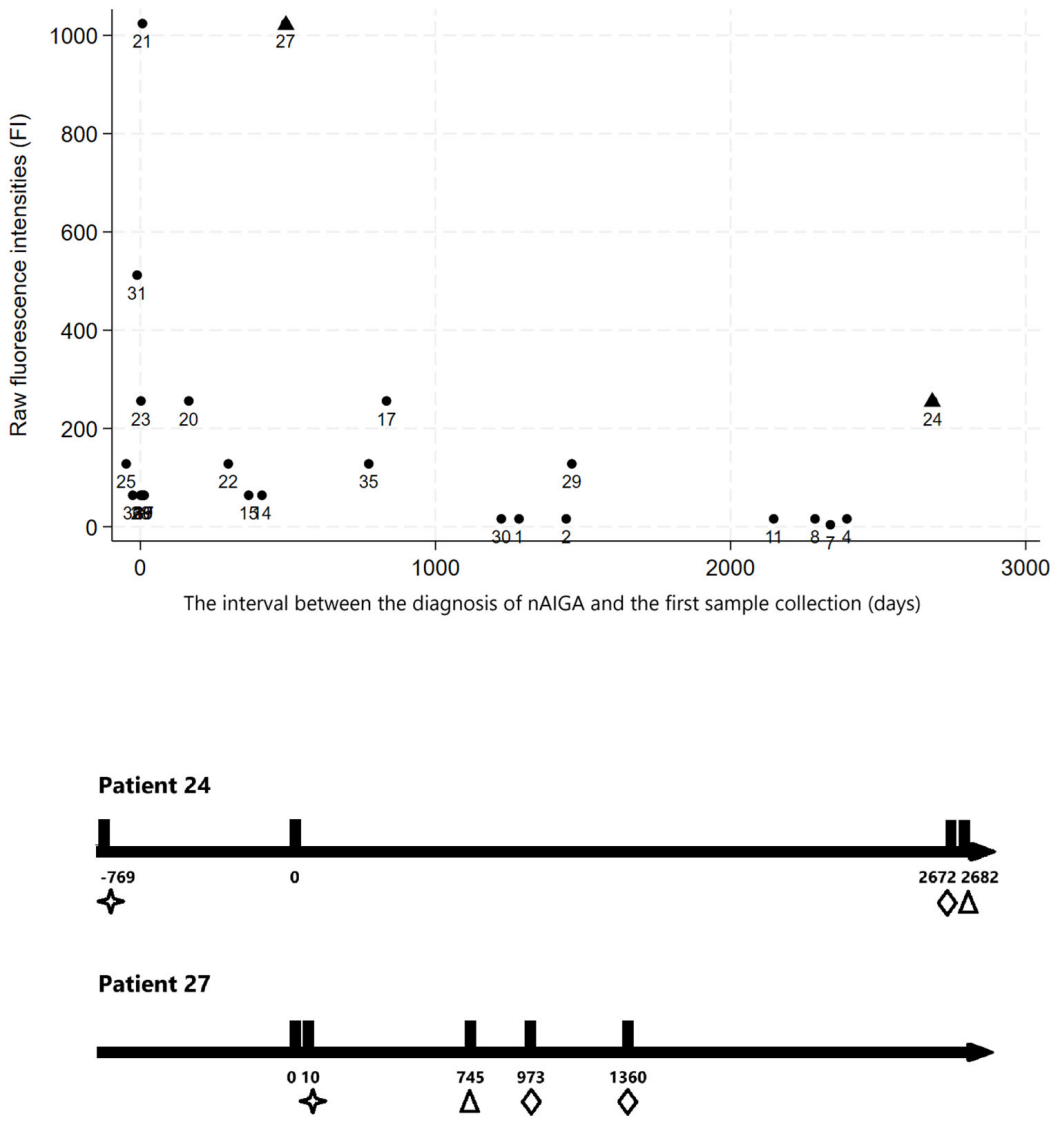
(Upper panel) The relationship between follow-up time and anti-IFN-γ binding titers of the first samples of the patients, expressed as raw fluorescence intensities (FI). First samples were obtained at different timepoints in relation to their disease onset; in general, the anti-IFN-γ binding titers of first samples obtained more than 1 year after disease onset were below 300, and more than 3 years after disease onset were below 200; (Lower panel) The follow-up timeline of the two outliers (days). We found two outliers (patient numbered 24 and 27, triangle on upper panel) with relatively rebound in anti-IFN-γ IgG fluorescence intensities during follow-up (hollow triangle marks point of testing of high binding titers on lower panel). The two individuals both had autoimmune diseases diagnosed (as indicated by hollow stars on lower panel) under immunosuppressant control. They developed incidental urothelial cell carcinoma, endometrial and breast cancers (as indicated by hollow diamonds) years after anti-IFN-γ associated immunodeficiency was diagnosed.

Follow-up duration and anti-IFN-γ binding titers were correlated in scatter plots, showing that titers generally decreased over time (**Figure 2A**). Patients numbered 24 and 27 were outliers with high anti-IFN-γ IgG binding titers. Their follow-up timeline was shown in **Figure 2B**. Patient numbered 24 was a woman diagnosed with autoimmune thyroiditis without maintenance immunosuppressant, who died of rapidly progressive urothelial cell carcinoma with disseminated metastases 9 years after nAIGA diagnosis. She also had breast microcalcifications. Patient numbered 27 with mixed connective tissue disease, autoimmune thyroiditis, and frequent mycobacteremia relapses, also developed endometrial cancer and breast cancer, 2.7 and 3.7 years respectively, after being diagnosed with nAIGA. Her endometrial cancer was first suspected due to an unusually persistent FDG-PET avid left inguinal lymph node and uterus despite diminishing uptake over other lymph nodes. Patient 19 had a papillary thyroid carcinoma that was also diagnosed by FDG-PET at onset of adult-onset immunodeficiency due to nAIGA; since thyroid hotspots are atypical sites of opportunistic infections. In contrast, one patient with biopsy-proven lung adenocarcinoma was downstaged from stage IV to IA2 about 2 years after cancer diagnosis. She received bone and lymph node biopsies due to the discrepant response of her primary lung lesion and bone and lymph nodes to target therapy. The bone lesions and lymph nodes initially attributed to cancer metastases were secondary to *M. szulgai* infection. These cases illustrated the utility of FDG-PET for monitoring therapeutic responses and differentiating infectious versus neoplastic lesions, among patients who have malignancies in the setting of nAIGA.

**Figure 3:**
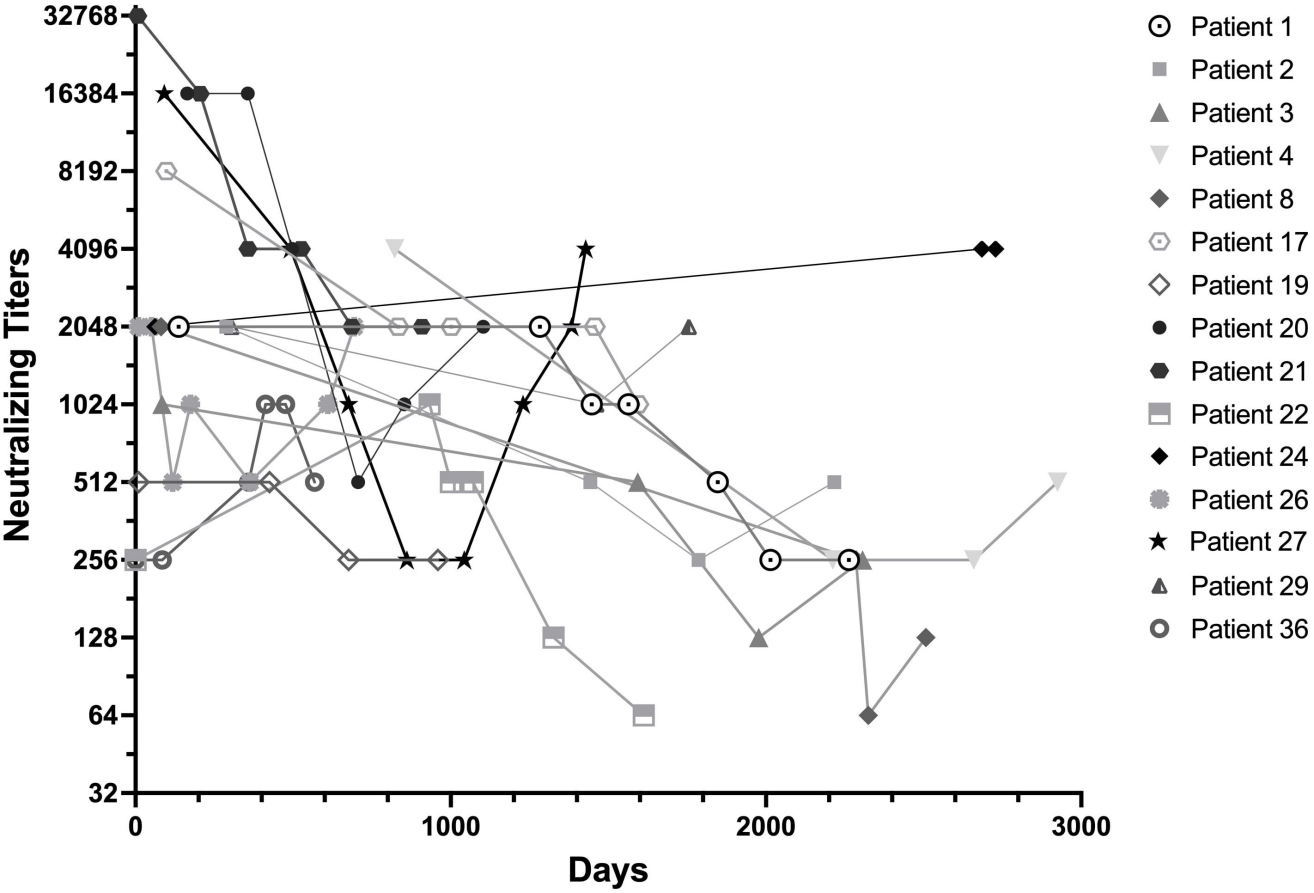
Longitudinal anti-IFN-γ neutralizing titers of the patients followed for more than 1,000 Days. Neutralizing titers were expressed as the first dilution of plasma that could no longer inhibit 1ng/mL of IFN-γ signaling. Most patients had decreased titers of neutralizing anti-IFN-γ activity over time, regardless of immunomodulation. Patient numbered 24, who despite being relapse-free and antimicrobial-free, had an upward trending increase in neutralizing anti-IFN-γ titers and was diagnosed with a rapidly progressive urothelial cancer 9 years after nAIGA diagnosis. She died within a year of cancer diagnosis. Patient numbered 27 despite initial reductions of neutralizing anti-IFN-γ secondary to infection control and rituximab, also had late upward trending neutralizing titers when she was sequentially diagnosed with endometrial and breast cancers.

In **Figure 3**, we tracked the longitudinal anti-IFN-γ neutralizing titers of 15 patients whose blood samples were banked and were followed for over 1,000 days. Patient numbered 24 also had an increasing trend in neutralizing antibody titers in addition to binding titers. Patient numbered 27 had rebound titers of neutralizing antibody despite corticosteroid, cyclophosphamide and rituximab at the time she developed endometrial and breast cancer.

A fourth (10/40) of the patients in this cohort were diagnosed with cancer; 4 before, 4 within one year, and 2 years after AOID diagnosis. The types of cancer in order of frequency were: papillary thyroid (2), hepatocellular (2), prostate (2), urothelial (1), endometrial (1), breast (1), non-small cell lung adenocarcinoma (1), Hodgkin’s lymphoma (1), and sigmoid colon (1) (two of the patients had double cancers).

## 4 Discussion

In this longitudinal cohort study of forty neutralizing anti-IFN-γ positive patients, the prevalence rate of nAIGA in patients with disseminated NTM infections was 63% during our study period, while it was 62-69% in previous studies (18, 19). Ninety-three percent of the patients were seropositive for one or more rheumatological markers, 24% had autoimmune thyroiditis, and 22% required immunomodulation. An increase in the number of autoimmune markers was associated with an increase in the incidence of nontuberculous mycobacteremia and mold infections and a trend towards increased mortality. Late-onset endometrial, breast and urothelial cell carcinomas were discovered among two female patients with extraordinarily high nAIGA titers that did not decline over time and papillary thyroid cancers were also diagnosed in two patients with autoimmune thyroiditis.

In the patients with Mendelian susceptibility to mycobacterial disease such as IFN-γ receptor deficiency, which is an inborn error of immunity with impaired Th1 cell and macrophage function, autoimmune diseases have not been described. This may be because patients with Mendelian susceptibility to mycobacterial disease die at a young age, leaving no time for autoimmunity to develop, and/or IFN-γ deficiency itself does not predispose to autoimmunity. However, anti-cytokine autoantibodies have been described in various autoimmune diseases such as RA, systemic lupus erythematosus (SLE), Sjogren’s syndrome, and systemic sclerosis (20, 21). For instance, anti-IFN-γ is associated with increased disease activity (21) and susceptibility to infections in SLE patients (22). Hence, it is clear that in patients with a genetic predisposition, autoimmunity can be induced by different T-cell epitopes and autoantibodies targeting nuclear antigens and cytokines can coexist. Although the origins of anti-IFN-γ autoantibodies remain unclear, the association with HLA-DR/DQ alleles implies impaired TCR-peptide-MHC interaction, which could subsequently lead to deficient thymic negative selection and downstream effects on the peripheral B cell tolerance. It is therefore unsurprising that in patients with nAIGA, loss of peripheral tolerance may also occur for other self-antigens, including nuclear antigens and organ-specific antigens.

Although thyroid autoantibodies are common in patients with nAIGA, there is no molecular mimicry between thyroid antigens and human IFN-γ. However, autoimmune thyroid destruction is driven primarily by Th1 immunity. Dendritic cells present immunogenic thyroid-related epitopes to autoreactive T cells and thyroid follicular cells, activating the IL-12/IFN-γ pathway (23). IL-12 and IL-23 can increase sodium iodine symporter (NIS) reporter gene expression, causing direct thyroid inflammation. IL-12 and IL23 stimulate IFN-γ secretion and subsequent secretion of CXCL9, CXCL10, and CXCL11 by thyroid follicular cells, where they interact with CXCR3 expressed by Th1 cells and create an amplification feedback loop (24). B cells also contribute to lymphocytic infiltration of the thyroid by producing autoantibodies against thyroid self-antigens (25). However, whether neutralizing anti-IFN-γ autoantibodies predate autoimmune thyroid autoantibodies or are produced in response to excessive IFN-γ-mediated inflammation is unclear. Plausibly, they may be produced as a mechanism to dampen the IFN-γ-mediated tissue destruction. In our cohort, six out of the nine patients with autoimmune thyroiditis developed autoimmune thyroiditis on average 993.9 days prior to nAIGA diagnosis. This finding supports the notion that anti-IFN-γ autoantibodies, which reduce IL-12/IL-23 levels, may also be produced to modulate ongoing IFN-γ production by thyroid follicular cells in patients with autoimmune thyroiditis (26).

Another possible mechanism is that, when peripheral tolerance fails, neutralizing anti-IFN-γ autoantibodies might develop along with other autoantibodies, in a milieu of inflammation and pro-survival factors for autoreactive B-cells. This collateral loss of self-tolerance can lead to concomitant autoimmune diseases such as chronic autoimmune gastritis, Sjogren syndrome, or rheumatoid arthritis (27). Our patients had a high incidence of gastroesophageal reflux disease and gastritis, although in most cases it was not ascertained whether this was autoimmune mediated.

Malignancies have been found in patients with IFN-γ receptor deficiency and impaired IFN-γ regulation of Th1 cell development. Cases of disseminated squamous cell carcinoma of the skin (28), EBV-related B-cell lymphoma (29), and pineal germinoma (30) have been reported in young patients. In our cohort, we also found four patients with genitourinary cancers, two of whom developed malignancies years after the diagnosis of adult-onset immunodeficiency (2672 and 973 days respectively). Interestingly, the nAIGA titers of patients with late-onset cancers showed an increase over time in spite of immunomodulatory agents, while a general decline is observed. In our cohort with a high background of autoimmune thyroiditis, two patients also developed papillary thyroid cancers, which supports the notion of malignancy arising from chronic inflammation. In addition, IFN-γ, TNF, interleukins, and other cytokines also modulates tumor biology (31). A high concentration of IFN-γ is associated with tumor suppression by inducing cancer cell apoptosis and inhibiting angiogenesis in the tumor microenvironment (32, 33). However, controversial findings have been reported in small studies on endometrial and urothelial bladder cancer (34, 35). Further mechanistic studies of the pathogenesis of specific cancers are needed if our results are validated. Preliminarily we would recommend to monitor neutralizing and binding anti-IFN-γ titers in order to ascertain their predictive value for late-onset cancers during long-term follow-up of patients with nAIGA.

Our study has several major limitations. First, the study is a single-center study with only 40 patients, limiting the interpretability of the associations and statistical analysis. Second, there was no universal screening for cancer; under-diagnosis is possible despite routine FDG-PET scans. Finally, the causal relationship between nAIGA and concomitant or subsequent autoimmune or neoplastic disease remains undetermined and these trends are anecdotal at best. However, as the terminal referral center in Taiwan with a de-emphasis on insurance-driven incentives, the care offered to these patients is comprehensive with few expenses spared, resulting in the highest possible level of retention and systematic, meticulous observations, that is rarely possible in other Asian countries where this condition is prevalent. A further multicenter cohort study is required to optimize the quality of care by establishing a protocol for anti-IFN-γ antibody titers, autoimmune markers, and malignancy screening.

## 5 Conclusions

Adult-onset immunodeficiency due to nAIGA is a syndrome with a high likelihood of concomitant autoimmunity. Chronic infection and autoimmune mediated inflammation may foster neoplastic changes, but the mechanism is still undetermined. Autoimmune disease and cancer surveillance for patients with long-term nAIGA is advised.

## Data Availability

The raw data supporting the conclusions of this article will be made available by the authors, without undue reservation.
