# Supplementary material for: Concomitant autoimmunity and late cancers in adult-onset immunodeficiency due to neutralizing anti-IFN-γ autoantibodies
Source: Front Immunol. 2025 Apr 17;16:1526439. doi: 10.3389/fimmu.2025.1526439 (PMC12043703; doi:10.3389/fimmu.2025.1526439)
Supplement: Supplementary file 1 [file Table1.docx]

**Supplementary tables**

**Supplementary Table 1.** Review of published reports on anti-IFN-γ patients’ infectious, autoimmune and neoplastic manifestations

|  |  | Opportunistic infections | Autoimmune or autoinflammatory disease | Malignancy |
| --- | --- | --- | --- | --- |
| Important cohort studies | Chi CY, 2016. (1) | Nontuberculous mycobacteria (n=46)  Varicella-zoster virus (n=28)  Non-typhoid Salmonella spp. (n=18) | Sjogren syndrome (n=1)  Bechet disease (n=1)  Hashimoto thyroiditis (n=1)  Autoimmune thyroiditis (n=1) | Peripheral T cell lymphoma (n=3)  Langerhan cell histiocytosis X (n=1) |
|  | Guo J, 2020. (2) | Talaromyces marneffei (n = 58)  Nontuberculous mycobacteria (n=7)  Mycobacterium tuberculosis (n=3)  Non-typhoid Salmonella spp. (n=4)  Varicella-zoster virus (n=8)  Cytomegalovirus (n=3)  Epstein-Barr virus (n=2)  Clonorchis sinensis (n=1)  Hepatitis B virus (n=2)  C. albicans (n=3) | None reported | None reported |
|  | Browne, S.K., 2012. (3) | Nontuberculous mycobacteria (estimated n=105)  Non-typhoid Salmonella spp. (estimated n=24)  Cryptococcus neoformans (estimated n=10)  Burkholderia pseudomallei (estimated n=4)  Histoplasma capsulatum (estimated n=7)  Penicillium marnefii (estimated n=7)  Varicella-zoster virus (estimated n=16)  Strongyloides stercoralis (estimated n=1) | Neutrophilic dermatosis (n=1) | None reported |
|  | Chi, C.Y., 2013. (4) | Nontuberculous mycobacteria (n=19)  Varicella-zoster virus (n=12)  Salmonella enteritidis (n=6)  Staphylococcus aureus (n=1)  Penicillium marneffei (n=2)  Cryptococcus neoformans (n=1)  Legionella spp. (n=1) | Erythema induratum (n=1) | None reported |
| Cases with autoimmune diseases | Döffinger R, 2004. (5) | Mycobacterium tuberculosis and Mycobacterium chelonae (n=1) | Autoimmune (type I) diabetes  Primary hypothyroidism (n=1) | None reported |
|  | Liu TT, 2016. (6) | Disseminated Mycobacterium abscessus lymphadenitis(n=1) | IgG4-related lymphadenopathy (n=1) | None reported |
|  | Patel SY, 2005. (7) | Nontuberculous mycobacteria (n=35)  Varicella-zoster virus (n=1) | Positive ANA (n=2), anti-Sm (n=1), anti-cardiolipin (n=1), anti-SSA (n=1), c-ANCA (n=1) | None reported |
| Case reports with Hematolo-gical cancers | Tanigaki T, 2022. (8) | Recurrent Mycobacterium avium complex infection (pulmonary, thoracic arthritis, pleurisy) (n=1)  Inguinal lymphadenitis with  Mycobacterium gordonae (n=1) | None reported | Intravascular large B-cell lymphoma 10 years after diagnosis of nAIGA |
|  | Koizumi Y, 2019. (9) | Disseminated Mycobacterium avium infection (peritoneal fluid, blood, lung) (n=1) | None reported | Angioimmunoblastic T-cell lymphoma |
| Case reports with  other atopic/  reactive dermatoses | Kantaputra, 2023. (10) | Nontuberculous mycobacteria (n=2) | Pustular eruption mimicking generalized pustular psoriasis (n=2) | None reported |
|  | Chan JF, 2013. (11) | Nontuberculous mycobacteria (n=3)  Talaromycosis (n=2)  Burkholderia pseudomallei (n=1) | Sweet syndrome (n=1)  Lobular panniculitis (n=1) | None reported |

**References:**

1. Chi CY, Lin CH, Ho MW, Ding JY, Huang WC, Shih HP, et al. Clinical manifestations, course, and outcome of patients with neutralizing anti-interferon-γ autoantibodies and disseminated nontuberculous mycobacterial infections. Medicine (Baltimore). 2016;95(25):e3927.

2. Guo J, Ning XQ, Ding JY, Zheng YQ, Shi NN, Wu FY, et al. Anti-IFN-γ autoantibodies underlie disseminated Talaromyces marneffei infections. J Exp Med. 2020;217(12):e20190502.

3. Browne SK, Burbelo PD, Chetchotisakd P, Suputtamongkol Y, Kiertiburanakul S, Shaw PA, et al. Adult-onset immunodeficiency in Thailand and Taiwan. N Engl J Med. 2012;367(8):725-34.

4. Chi CY, Chu CC, Liu JP, Lin CH, Ho MW, Lo WJ, et al. Anti-IFN-γ autoantibodies in adults with disseminated nontuberculous mycobacterial infections are associated with HLA-DRB1*16:02 and HLA-DQB1*05:02 and the reactivation of latent varicella-zoster virus infection. Blood. 2013;121(8):1357-66.

5. Döffinger R, Helbert MR, Barcenas-Morales G, Yang K, Dupuis S, Ceron-Gutierrez L, et al. Autoantibodies to interferon-gamma in a patient with selective susceptibility to mycobacterial infection and organ-specific autoimmunity. Clin Infect Dis. 2004;38(1):e10-4.

6. Liu TT, Weng SW, Wang MC, Huang WT. Nontuberculous mycobacterial infection with concurrent IgG4-related lymphadenopathy. Apmis. 2016;124(3):216-20.

7. Patel SY, Ding L, Brown MR, Lantz L, Gay T, Cohen S, et al. Anti-IFN-γ Autoantibodies in Disseminated Nontuberculous Mycobacterial Infections. The Journal of Immunology. 2005;175(7):4769-76.

8. Tanigaki T, Kimizuka Y, Maki Y, Sato C, Yoshimatsu S, Ogata H, et al. Development of intravascular large B-cell lymphoma during prophylactic antibiotic treatment for anti-interferon-gamma autoantibody syndrome: A case report. J Infect Chemother. 2022;28(11):1562-6.

9. Koizumi Y, Sakagami T, Minamiguchi H, Makino A, Aoki A, Hodohara K, et al. Chylous ascites, anti-interferon-gamma autoantibody, and angioimmunoblastic T-cell lymphoma: a rare but intriguing connection over Mycobacterium avium. Med Microbiol Immunol. 2019;208(1):33-7.

10. Kantaputra P, Daroontum T, Chuamanochan M, Chaowattanapanit S, Kiratikanon S, Choonhakarn C, et al. SERPINB3, Adult-Onset Immunodeficiency, and Generalized Pustular Psoriasis. Genes (Basel). 2023;14(2):266.

11. Chan JF, Trendell-Smith NJ, Chan JC, Hung IF, Tang BS, Cheng VC, et al. Reactive and infective dermatoses associated with adult-onset immunodeficiency due to anti-interferon-gamma autoantibody: Sweet's syndrome and beyond. Dermatology. 2013;226(2):157-66.

**Supplementary Table 2.** Initial immune and laboratory data at time of diagnosis of adult-onset immunodeficiency associated with neutralizing anti-IFN-γ autoantibodies (AIGA) or concomitant autoimmune markers

| Variables | | n | | Total | | Reference range | |
| --- | --- | --- | --- | --- | --- | --- | --- |
| Initial immune profiles on the diagnosis of AIGA, median (IQR) | | | | | | | |
| Total lymphocyte count, cells/ul | | 23 | | 2111 (1422-2775) | | 1052-3182 | |
| CD3+ lymphocyte count, cells/ul | | 21 | | 1231 (967-1782) | | 672-2366 | |
| CD4+ lymphocyte count, cells/ul | | 21 | | 601 (506-944) | | 292-1366 | |
| CD8+ lymphocyte count, cells/ul | | 21 | | 589 (440-774) | | 240-1028 | |
| CD19+ lymphocyte count, cells/ul | | 21 | | 158 (96-323) | | 82-560 | |
| CD16+56+ lymphocyte count, cells/ul | | 15 | | 370 (184-584) | | 130-938 | |
| IgG, mg/dL | | 28 | | 1735.5 (1490-2180) | | 635-1741 | |
| IgG-1, mg/dL | | 15 | | 1140 (935-1440) | | 405-1011 | |
| IgG-2, mg/dL | | 14 | | 451.5 (281-537) | | 169-786 | |
| IgG-3, mg/dL | | 13 | | 77.2 (53-97) | | 11-85 | |
| IgG-4, mg/dL | | 16 | | 130 (48-208) | | 3-201 | |
| IgM, mg/dL | | 24 | | 118.1 (78-158) | | 45-281 | |
| Initial laboratory findings on the detection of autoimmune markers, median (IQR) | | | | | | | |
| White blood cell, K/μL | | 31 | | 13 (8.7-21.2) | | 3.54-9.06 | |
| Haemoglobin, g/dL | | 32 | | 9 (8.4-11.8) | | 13.2-17.2 | |
| Platelet, K/μL | | 32 | | 404 (322-511) | | 148-339 | |
| C3, mg/dL | | 23 | | 128 (106.1-155.5) | | 87-200 | |
| C4, mg/dL | | 23 | | 40 (30.1-48.0) | | 19-52 | |
| D-dimer, mg/L FEU | | 17 | | 2.1 (0.9-2.9) | | ≤0.56 | |
| ESR, mm/hr | | 19 | | 70 (20-92) | | 2-15 | |
| CRP, mg/dL | | 27 | | 5.5 (2.3-10.6) | | <1 | |
| Ferritin, ng/mL | | 19 | | 441 (109.3-692) | | 21.81-274..66 | |
| IgG, cells/ul | | 28 | | 2050 (1670-2600) | | 635-1741 | |

Abbreviations: AIGA, anti-interferon--γ autoantibodies; CRP, c-reactive protein; ESR, erythrocyte sedimentation rate; IgG, immunoglobulin G; IgM, immunoglobulin M.
